# Tumor Necrosis Factor-Alpha Inhibitors and Cardiovascular Risk in Rheumatoid Arthritis: A Systematic Review

**DOI:** 10.7759/cureus.26430

**Published:** 2022-06-29

**Authors:** Shaalina Nair, Simranjit Singh Kahlon, Rabia Sikandar, Aishwarya Peddemul, Sreedevi Tejovath, Danial Hassan, Khushbu K Patel, Jihan A Mostafa

**Affiliations:** 1 Internal Medicine, California Institute of Behavioral Neurosciences & Psychology, Fairfield, USA; 2 Obstetrics and Gynecology, California Institute of Behavioral Neurosciences & Psychology, Fairfield, USA; 3 Department of Healthcare Professions, Ministry of Public Health, Doha, QAT; 4 Cardiology, California Institute of Behavioral Neurosciences & Psychology, Fairfield, USA; 5 Psychiatry, California Institute of Behavioral Neurosciences & Psychology, Fairfield, USA

**Keywords:** rheumatoid arthritis, congestive heart failure, cerebrovascular diseases, myocardial ischemia and infarction, reduce the risk of coronary artery disease in rheumatoid arthritis, risk of cardiovascular diseases, tumor necrosis factor-alpha (tnf-α) inhibitors

## Abstract

Rheumatoid arthritis (RA) is an autoimmune disease that, if untreated or poorly controlled, can cause significant morbidity in terms of loss of physical function and higher mortality due to higher cardiovascular risk. The standard of care for this disease is the use of disease-modifying antirheumatic drugs (DMARDs). However, patients unable to reach low disease activity or remission and patients unable to tolerate conventional DMARDs will be switched to biologic therapy, a subset of which includes anti-tumor necrosis factor-alpha inhibitors. Since tumor necrosis factor-alpha inhibitors (TNFi) inhibit the inflammatory cascade, they also play an essential role in dampening the progression of atherosclerosis and altering the risk of cardiovascular outcomes in RA.

In this study, we assessed the risk of cardiovascular diseases, namely, congestive heart failure, nonfatal myocardial infarction, cerebrovascular disease, and coronary artery disease. We carried out the analysis by following the Preferred Reporting Items for Systematic Reviews and Meta-Analyses (PRISMA) guidelines and conducted a literature search utilizing the following databases: PubMed, Science Direct, and Cochrane Library. Using the search strategy, we found a total of 19 articles that fit the inclusion and exclusion criteria, in addition to passing the risk of bias assessment. This is composed of three systematic reviews with meta-analyses, three randomized control studies, four narrative reviews, and nine cohort studies. In this systematic review, it was found that treatment with TNFi causes a corresponding reduction in the risk of cardiovascular events. This review encourages further dissection into the inner workings of TNFi in reducing the risk of cardiovascular disease among patients with RA.

## Introduction and background

Rheumatoid arthritis (RA) is part of a spectrum of systemic inflammatory arthropathies with a lifetime prevalence of 1% worldwide [1]. It is an autoimmune disorder characterized by the presence of autoantibodies, such as rheumatoid factor (RF) and anti-cyclic citrullinated peptide (anti-CCP), which are often formed for many years in a patient before detection of the disease [2]. As per the guidelines published by the American College of Rheumatology/European League Against Rheumatism collaborative initiative in 2010, a diagnosis of "definite RA" is based on the confirmed presence of synovitis in at least one joint, absence of an alternative diagnosis that better explains the synovitis, and achievement of a total score of 6 or greater (of a possible 10) from the individual scores in four domains: number and size of involved joints, serologic abnormality, elevated acute-phase response, and symptom duration [3].

Pharmacologic management of patients with RA includes disease-modifying antirheumatic drugs (DMARDs), anti-tumor necrosis factor-alpha inhibitors, such as infliximab, etanercept, and adalimumab, and non-tumor necrosis factor inhibitors such as abatacept, rituximab, and tocilizumab [4]. The chronic state of inflammation and the subsequent inflammatory response has led to an increased risk of cardiovascular disease (CVD). CVD mortality is increased by approximately 50% in RA patients compared with the general population [5]. The pro-inflammatory cytokine tumor necrosis factor-alpha (TNF-alpha) and C-reactive protein (CRP) may play an important role in accelerating the progression of CVD [6]. This is caused by the increased rate of atherosclerosis present in RA precipitated by its chronic inflammatory state [7]. Furthermore, several studies have shown that the use of tumor necrosis factor-alpha inhibitors (TNFi) in patients with RA has led to improvement in CVD with primary endpoints being congestive heart failure, nonfatal myocardial infarction, and lipid profiles [8-10].

This systematic review highlights the role of TNFi in reducing CVD risk in RA patients. Multiple randomized clinical trials, meta-analyses, cohort studies, and systematic reviews have been published on the topic mentioned; however, there still exists an element of uncertainty regarding the ability of such a drug to feasibly reduce CVD risk. This review aims to identify and present the latest information and data about the causal relationship between the use of TNFi and the reduction of CVD risk in RA. By justifying such a relationship, this study can open up more avenues for further trials and the use of TNFi to further impair the progression of CVD in RA patients.

## Review

Methods

In this section, the criteria for inclusion and exclusion, the search strategy employed, the ascertainment of risk of bias in individual studies, and the selection of studies will be explained in detail.

*Inclusion and Exclusion *Criteria

The inclusion criteria used for this systematic review are outlined in Table 1 below. The exclusion criteria are studies that were case reports, case series, abstracts presented at conferences, cross-sectional studies, and studies that did not fit all the inclusion criteria listed below in Table 1.

**Table 1:** Inclusion criteria

Type of studies	Study subject criteria	Cardiovascular condition of interest
Randomized clinical trials, non-randomized clinical trials, cohort studies, case-control studies, traditional reviews, and systematic reviews	Adult population age > 30 years old who are diagnosed with rheumatoid arthritis with tumor necrosis factor-alpha inhibitors as part of current medication regime either solitary or in combination with other disease-modifying antirheumatic drugs	Congestive heart failure, nonfatal myocardial infarction, cerebrovascular disease (ischemic stroke and transient ischemic attack), and coronary artery disease

Search Strategy

To obtain research articles relevant to the topic, three research databases were thoroughly utilized and included PubMed, Science Direct, and Cochrane Library. All databases were last accessed on January 28, 2021. The search was done using regular keywords and MeSH (Medical Subject Heading) keywords according to the type of database used, as showcased in Table 2.

**Table 2:** Search strategy employing various databases

Type of database	Keywords	Filter criteria	Search results
PubMed	Tumor necrosis factor-alpha/antagonists inhibitors [Majr] OR tumor necrosis factor-alpha/therapeutic use [Majr] AND cardiovascular diseases [Majr] AND arthritis, rheumatoid [Majr]	Article types: clinical trials, randomized clinical trials, meta-analysis, systematic review, and review articles. Publication dates: 2002-2022	92
Science Direct	Tumor necrosis factor inhibitors AND cardiovascular disease AND rheumatoid arthritis	Article types: review articles and research articles. Subject areas: medicine and dentistry. Publication dates: 2002-2022	3757
Cochrane Library	Tumor necrosis factor inhibitors AND cardiovascular disease AND rheumatoid arthritis	Article types: clinical trials. Publication dates: 2005-2022	18

The references that were obtained from databases were sorted alphabetically using Microsoft Excel 2021 (Microsoft Corporation, Redmond, WA) for duplicates removal. The studies were then screened thoroughly using titles and abstracts to filter out studies that did not meet the inclusion criteria. The records were reviewed based on the titles and abstracts, excluding irrelevant studies. After reviewing, a retrieval of the full-text articles followed this. Study protocols, case reports, and conference abstracts were excluded from this review due to the lower yield of material published.

Ascertainment of Risk of Bias in Individual Studies

All articles that met the inclusion criteria were subjected to risk of bias ascertainment using tools that were specific for the original study type. Cochrane Collaboration Risk of Bias Tool (CCRBT) was used for randomized clinical trials (RCTs), Newcastle Ottawa Scale (NOS) for cohort studies and non-randomized clinical trials (NRCTs), Assessment of Multiple Systematic Reviews 2 (AMSTAR 2) for systematic reviews and meta-analyses, and Scale for the Assessment of Narrative Review Articles 2 (SANRA 2) for narrative reviews [11-14]. A score of at least 70% for each assessment tool was used as the benchmark of acceptance of the study (Table 3), which has been adapted from a study by Yu et al. [15].

**Table 3:** Risk of bias assessment AMSTAR 2: Assessment of Multiple Systematic Reviews 2; SANRA 2: Scale for the Assessment of Narrative Review Articles 2; CCRBT: Cochrane Collaboration Risk of Bias Tool; NOS: Newcastle-Ottawa Scale; RCTs: randomized clinical trials; NRCTs: non-randomized clinical trials.

Quality assessment tool	Study type	Total score	Accepted score > 70%	Studies accepted
AMSTAR 2 [11]	Systematic reviews and meta-analyses	16	12	Peters et al. [16], Barnabe et al. [17], Westlake et al. [18]
SANRA 2 [12]	Narrative reviews	12	9	Cacciapaglia et al. [19], Jarlborg et al. [20], Ferraccioli [21], Szekanecz et al. [22]
CCRBT [13]	RCTs	7	5	Weisman et al. [23], Giles et al. [24], Solomon et al. [25]
NOS [14]	Cohort studies and NRCTs	8	6	Setoguchi et al. [26], Al-Aly et al. [27], Bili et al. [28], Desai et al. [29], Dixon et al. [30], Jin et al. [31], Kang et al. [32], Low et al. [33,34]

Selection of Studies

In regards to the selection of studies, two authors (Shaalina Nair and Simranjit Kahlon) did an initial screening of all articles that met the inclusion criteria by reviewing their titles and abstracts. The articles in English and only original studies that involved RA patients currently receiving TNFi therapy that also reported on cardiovascular events were accepted. The studies that were found relevant to the study question were further dissected in the full-text review in the second stage of screening. Systematic reviews and meta-analyses, narrative reviews, RCTs, cohort studies, and NRCTs that satisfied the inclusion criteria and passed the quality assessment tools with low risk of bias then underwent full-text review. Articles that were included in this systematic review must have reported original data on RA patients receiving TNFi therapy compared to patients receiving DMARD therapy or other biological therapies and described cardiovascular events including all events, myocardial infarction (MI), congestive heart failure (CHF), cerebrovascular accident (CVA), and coronary artery disease (CAD).

Results

**Figure 1:**
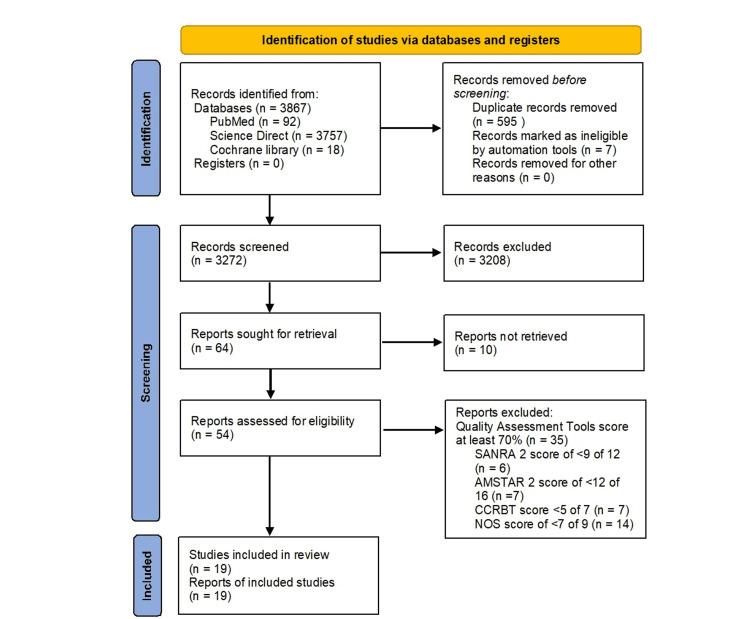
Identification of studies via databases and registers CCRBT: Cochrane Collaboration Risk of Bias Tool; NOS: Newcastle-Ottawa Scale; AMSTAR 2: Assessment of Multiple Systematic Reviews 2; SANRA 2: Scale for the Assessment of Narrative Review Articles 2.

This systematic review was conducted based on the Preferred Reporting Items for Systematic Reviews and Meta-Analyses (PRISMA) 2020 guidelines and the search strategy is outlined in Figure 1 below [35].

A total of 3867 studies were obtained after searching using three databases, namely, PubMed, Science Direct, and Cochrane Library, using the search strategy described. Of the 3867 studies, 3272 articles were screened after duplication removal and removal of records marked ineligible. Furthermore, 3208 articles were found to fit the exclusion criteria and were excluded with 10 articles not retrieved from the databases. This left us with the remaining 54 articles that were put through eligibility assessment. Out of 54 articles, only 19 articles managed to match the inclusion criteria of this study and were included in this systematic review, characteristics of which are outlined in Table 4 below, which is adapted from a study by Barnabe et al. [17]. In summary, it has been identified that three systematic reviews with meta-analyses, four narrative reviews, three RCTs, and nine cohort studies passed the risk of bias ascertainment process and inclusion criteria of this study.

**Table 4:** Overview and summary of randomized clinical trials and cohort studies RCT: randomized clinical trial; TNFi: tumor necrosis factor-alpha inhibitor; CVD: cardiovascular disease; CHF: congestive heart failure; RA: rheumatoid arthritis; DMARD: disease-modifying antirheumatic drug.

Reference/article	Article type	Exposure - TNFi therapy used	CVD outcome	Total number of cases	Duration of disease	Follow-up time	Conclusion
Weisman et al. [23]	RCT	Etanercept subcutaneous (SC) 25 mg twice weekly for 16 weeks or SC injections of placebo	Severe cardiovascular disease (CVD) event (heart failure, coronary artery disease, MI, and cerebrovascular disease	266 cases with a mean age of 60.6 years and 72.2% female, 269 controls (placebo ± methotrexate (MTX)) with a mean age of 59.3 years, and 78.1% female	Mean of 9.4 years for cases and 10.1 years for controls	16 weeks	No significant risk of increased CVD events with etanercept vs. placebo ± MTX group
Giles et al. [24]	RCT	Intravenous tocilizumab (8 mg/kg every 4 weeks) or SC etanercept (50 mg weekly)	Major adverse CVD events (MACE) include cardiovascular-related death, nonfatal myocardial infarction, and nonfatal stroke (of any type)	1538 cases were randomly assigned to the tocilizumab group with a mean age of 61 ± 7 years and 77.6 % female with 1542 controls randomly assigned to the etanercept group and with mean age of 61 ± 8 years and 77.9% female	≥6 months for both cases and controls	Mean of 3.2 years	The risk of MACE in patients treated with tocilizumab is 5% higher than in patients treated with etanercept
Solomon et al. [25]	RCT	Adalimumab, etanercept, and infliximab	A new incidence of myocardial infarction, stroke, or coronary revascularization	11,587 cases using TNFi therapy with a mean age of 55.4 years and 86.5% female, with a history of myocardial infarction, stroke, coronary revascularization, and CV risk factors. 8656 controls using non-biologic disease-modifying anti-rheumatic drugs (nbDMARDS) like hydroxychloroquine leflunomide or sulfasalazine with a mean age of 56.2 years and 85.9% female	Not stated	1 year	In the first 6 months of follow-up, the hazard ratio was 0.8 in the treatment group compared to the control group. Therefore, the cardiovascular risk was lower among the users of TNF inhibitors (TNFi) in comparison with DMARDs
Setoguchi et al. [26]	Cohort study	Etanercept, infliximab, and adalimumab	Incidence of heart failure (HF) hospitalization, risk of HF hospitalization, and risk of death among patients with the previous CHF	1002 cases using TNFi therapy with mean age of 73 years and 99.9% females. 998 controls using MTX with mean age of 77 years and 99.9% females. Both cases and controls were further divided into groups who had previous HF or do not have previous HF	2 years	24 months	Incidence of HF hospitalization is increased in TNFi therapy users (1.43 for the group without a history of HF and 1.39 for the group with previous HF). There is also an increased risk of HF hospitalization in patients with and without a history of HF who use TNFi. There is a 4.2-fold increase in the risk of death in patients who use TNFi compared to MTX
Al-Aly et al. [27]	Cohort study	Etanercept, infliximab, and adalimumab	Time from study entry to the occurrence of cardiovascular events, which include atherosclerotic heart disease, congestive heart failure, peripheral artery disease, and cerebrovascular disease	3,796 cases using TNFi therapy with a mean age of 57 ± 12 years and 9% females. 19,899 controls using MTX with a mean age of 63 ± 12 years and 9% females	≥4 months for both cases and controls	Average of 842 days for cases and 1128 days for controls	Long-term exposure to TNFi therapy had no significant effect on combined cardiovascular outcomes or all-cause mortality. There is a decreased risk of cardiovascular events associated with the use of TNFi in cases who were younger than 63 years old with a concomitant decrease in risk of cerebrovascular disease
Bili et al. [28]	Cohort study	Etanercept, adalimumab, infliximab, golimumab, and certolizumab	The primary outcome is incident coronary artery disease (CAD) defined as MI, unstable angina, or cardiac revascularization procedure. The secondary outcome was adjudicated incident CVD, defined as a composite of CAD, stroke, transient ischemic attack, abdominal aortic aneurysm, peripheral arterial disease, or arterial revascularization procedure	1022 cases were included in this study, 72.8% were women with a mean age at RA diagnosis of 51.7 ± 13 years. There were two reference groups: MTX and other non-biologic DMARDs. 1698 patients were included in the MTX group with a mean age of 56.2 ± 14 years and 72.8% female. 898 patients were included in the non-biologic DMARD group with a mean age of 56.9 ± 14 years and 74.8% female	Not stated	Median of 3.4 years	TNFi use is associated with decreased incidence of CAD in patients with RA and no previous cardiovascular disease (CVD). Use of TNF inhibitors for >16.1 months was associated with a relative risk for CAD of 0.18 (95% CI: 0.06-0.50) and CVD of 0.31 (95% CI: 0.15-0.65) compared to the reference group
Desai et al. [29]	Cohort study	Adalimumab, certolizumab, etanercept, golimumab, and infliximab	Cardiovascular (CV) event that consists of acute myocardial infarction, unstable angina, angina pectoris, CHF, and cerebrovascular disease	279 cases with incident CV disease and 3384 controls. Cases and matched controls (non-biologic DMARDs) were 64 years old at the index date, and 65.2% of the cases and controls were women	≥1 year	Mean of 238 days	The adjusted risk of CV events was not significantly different between patients who use TNFi therapy and non-biologic DMARD (incidence rate ratio 0.92, 95% CI: 0.59-1.44)
Dixon et al. [30]	Prospective cohort study	Etanercept, infliximab, and adalimumab	Rate of myocardial infarction (MI)	8670 cases treated with TNFi therapy and 2170 controls treated with traditional DMARDs	≥6 months for both cases and controls	Median of around 2 years	No significant decrease in the rate of MI in patients using TNFi compared to DMARDs (incidence rate ratio: 1.44; 95% CI: 0.56-3.67). The risk of MI is markedly reduced in those who respond to anti-TNF-alpha therapy by 6 months compared with non-responders
Jin et al. [31]	Cohort study	Adalimumab, etanercept, certolizumab, golimumab, and infliximab	A composite CVD endpoint including MI, stroke/transient ischemic attack (TIA), or coronary revascularization	6102 cases from the Medicare database who are diagnosed with RA and currently treated with abatacept (ABA) or TNFi therapy with a mean age of 73.8 years and around 83% female. 6934 controls from the MarketScan database who are diagnosed with RA and currently treated with abatacept or TNFi therapy with a mean age of 56.9 years and 82% females	≥1 year	6 years	The risk of a composite CVD endpoint was lower in cases from Medicare who are taking ABA compared with those who initiated on TNFi compared to the MarketScan group. There was no association between ABA and CVD risk
Kang et al. [32]	Cohort study	Adalimumab, certolizumab, etanercept, golimumab, and infliximab	The cardiovascular endpoint of myocardial infarction (MI), stroke/transient ischemic attack, and coronary revascularization	11,264 subjects from the Medicare database who are diagnosed with RA and currently treated with abatacept (ABA) or TNFi therapy with a mean age of 73.8 years and around 78% female from the diabetes mellitus (DM) subgroup. 12,434 subjects from the MarketScan database who are diagnosed with RA and currently treated with abatacept (ABA) or TNFi therapy with a mean age of 59 years and around 76% female from the non-DM subgroup. 20,635 subjects from the Medicare database who are diagnosed with RA and currently treated with abatacept (ABA) or TNFi therapy with a mean age of 73.3% years and around 81.9% female from the DM subgroup. 59,972 subjects from the MarketScan database who are diagnosed with RA and currently treated with abatacept (ABA) or TNFi therapy with a mean age of 54 years and around 79.2% female from the non-DM subgroup	Not stated	Average of 410 days in the DM subgroup and an average of 455 days in the non-DM subgroup	The risk of CVD endpoint was lower in ABA versus TNFi in the DM subgroup, with a pooled HR of 0.74 (95% CI, 0.57-0.96), but not in the non-DM subgroup, with a pooled HR of 0.94 (95% CI, 0.77-1.14)
Low et al. [33]	Cohort study	Adalimumab, etanercept, or infliximab	Risk of ischemic stroke and all stroke subtypes, variation in risk of ischemic stroke over time, and all-cause mortality after first ischemic stroke	11,642 cases received TNFi therapy with a mean age of 56.0 ± 12.2 years and 76.5% female. 3271 controls received synthetic DMARDs with a mean age of 59.9 ± 12.3 years and 73.5% female	Not stated	4-6 years	No significant association between exposure to TNFi and ischemic stroke (hazard ratio: 0.99; 95% CI: 0.54-1.81). Mortality 30 days or 1 year after ischemic stroke was not associated with concurrent TNFi exposure (odds ratio: 0.18; 95% CI: 0.03-1.21 and 0.60; 95% CI: 0.16-2.28, respectively)
Low et al. [34]	Cohort study	Adalimumab, etanercept, or infliximab	Myocardial infarction severity and mortality	11,200 cases received TNFi therapy with a mean age of 55.6 ± 12.3 years and 78% female. 3058 controls received synthetic DMARDs with a mean age of 59.9 ± 12.5 years and 75% female	Not stated	Median of 3.5 years and 5.3 years in cases and controls, respectively	A 39% reduction in the risk of MI was observed in patients treated with TNFi compared with those on synthetic DMARD therapy. The severity of MI and mortality post-MI were not associated with TNFi therapy

Among the three systematic reviews, one review explained the changes in lipid levels with TNFi therapy while the remaining two reviews explained the association between TNFi therapy and MI, CHF, and CVA. Furthermore, among the four narrative reviews, one review explained the association between TNFi therapy and CHF, one review explained the systemic effects of interleukin-6 (IL-6) and its subsequent blockade leading to cardiovascular disease risk, one review narrated the mechanism behind increased levels of anticardiolipin antibodies with TNF-alpha blockade leading to increased CVD, and one review mentioned in detail the effect of using TNFi therapy on the mechanism of atherosclerosis. In addition, three RCTs were selected for this systematic review, which include one RCT explaining the role of TNFi therapy in decreasing aortic stiffness by reducing aortic inflammation, one RCT providing insights into the CV safety of tocilizumab as compared to etanercept, and one RCT mentioning regarding the relationship between TNFi therapy and MI, stroke, or coronary revascularization after six months. The RCTs and the remaining 10 cohort studies are summarized in Table 4.

Discussion

The systematic review strongly upholds the hypothesis with provisions that treatment with TNFi causes a corresponding reduction in risk of cardiovascular events, namely, MI, stroke, transient ischemic attack (TIA), and CAD. This is supported by a decreased risk of cardiovascular (CV) events exhibited by more than half of the cohort studies and two out of three RCTs. For heart failure, only one study compared tumor necrosis factor (TNF) antagonist use with methotrexate (MTX) monotherapy and this demonstrated an increased risk in the TNF antagonist users.

Tumor Necrosis Factor Inhibitors Use and Their Relationship With Atherosclerosis

As part of the systemic inflammation cascade in patients with RA, macrophage-derived inflammatory cytokines and adipokines, for example, IL-6, TNF, interleukin-1β (IL-1β), and resistin, are overexpressed and represent critical points for effective treatment of the signs and symptoms of the disease [35]. Atherosclerosis represents a buildup of minor insults to the blood vessels leading to the formation of atheroma and subsequent thrombus if a plaque ruptures. It comprises a triad that includes endothelial dysfunction, dyslipidemia, and coagulation cascade activation.

These cytokines and adipokines play an important role in creating long-term changes in vasculature and speeding up the process of atherosclerosis [36]. One such example of long-term change to the vasculature is by the cytokine TNF-alpha. This cytokine is believed to encourage the apoptosis of endothelial cells, and with persistently active synthesis and release of TNF-alpha in the bloodstream, there will be continuous vascular damage [37].

Early vascular changes in RA can be detected with surrogate markers of atherosclerosis, which include common carotid intimal‑medial thickness (CCIMT), endothelial dysfunction, indicated by impaired flow-mediated vasodilatation (FmD) of the brachial artery, and arterial stiffness, which has been mentioned in several studies [38-40]. Endothelial dysfunction specified by FmD of the brachial artery has been shown in a study by Wållberg-Jonsson et al. that it often precedes atherosclerosis in patients with RA [41]. TNFi has been shown to improve FmD with reduced levels of CRP after 12 weeks of treatment [42]. On the other hand, evidence is rather conflicting regarding the effects of TNFi on CCIMT and arterial stiffness in patients with inflammatory rheumatic diseases [22].

**Figure 2:**
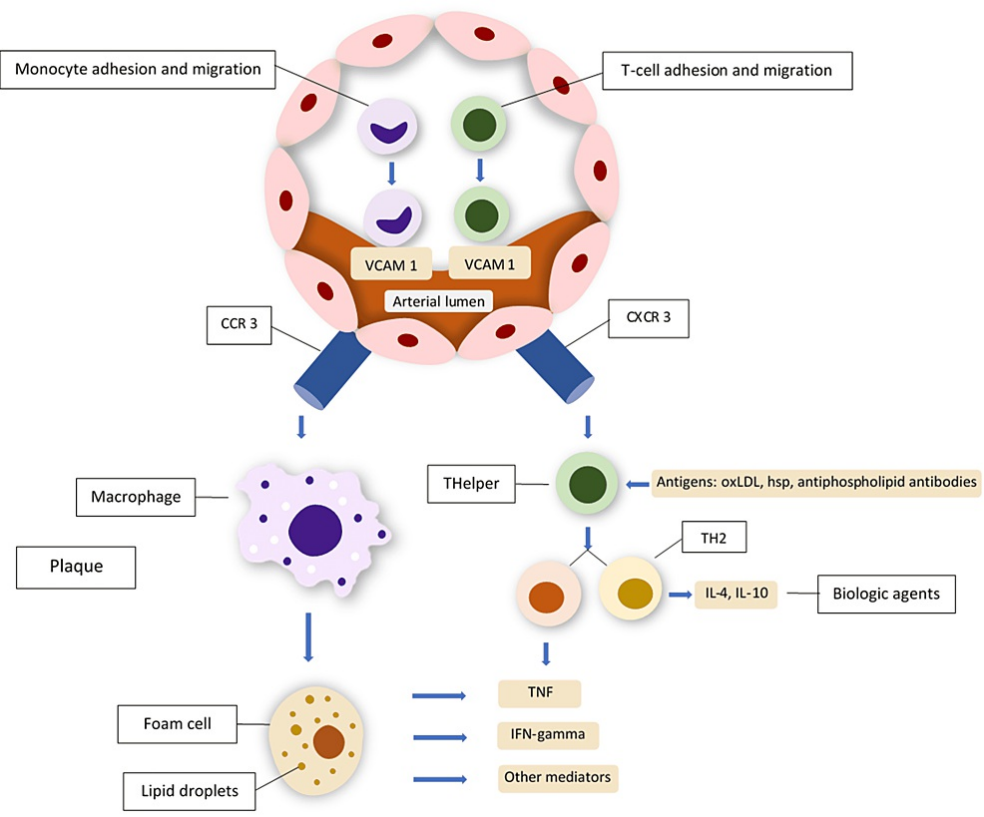
Inflammatory events outlined in atherosclerosis Monocytes pass through the arterial wall and then express the CCR3 chemokine receptor and bind chemokines. Monocytes then differentiate into macrophages and then foam cells, which are the main components in atherosclerotic plaque formation. T lymphocytes also move into the vessel wall, where they express chemokine receptors and differentiate into TH1 and TH2 cells. Both macrophages and TH1 cells release, among other inflammatory mediators, TNF. Therefore, TNFi biologic agents may be effective in controlling atherosclerosis related to rheumatoid arthritis. CCR3: CC‑chemokine receptor 3; CXCR3: CXC‑chemokine receptor 3; HSP: heat shock proteins; IFN: interferon; IL: interleukin; oxLDL: oxidized low‑density lipoprotein; TH: T‑helper lymphocyte; TNF: tumor necrosis factor; vCAM‑1: vascular cell adhesion molecule 1. Adapted from and used with permission from Professor Dr. Szekanecz Zoltan [22].

Another important point to note is that the rate of atherosclerosis can be reduced with the usage of TNFi, and its pathophysiology involves interactions between two key lipokines, namely, resistin and adiponectin, as detailed in a study by Kapoor [43]. Resistin is pro-atherogenic whereas adiponectin is anti-atherogenic. Although low levels of circulating adiponectin have been associated with endothelial dysfunction, as well as with RA, attenuation in resistin production using TNFi was associated with a reduction in CRP levels. This concludes that biological treatment can alter a person's atherogenic profile. The inflammatory pathways involved in atherosclerosis are summarized in Figure 2 below.

The Effect of Tumor Necrosis Factor Inhibitors on Long-Term Cardiovascular Complications

It is widely known and accepted that chronic inflammatory conditions such as RA do lead to long-term CVD. Data from several studies have outlined the four major factors that contribute to CV risk in RA. Firstly, the traditional CV risk factors include smoking, dyslipidemia, hypertension, obesity, and DM, which often coexist with a higher rate in patients with RA [44,45]. Secondly, the administration and use of glucocorticoids and non-steroidal anti-inflammatory drugs [46,47]. The third would be the existence of anti-citrullinated peptide antibodies (ACPAs) and rheumatoid factors (RFs), which are independent risk factors for CVD and CV mortality [48,49]. Lastly, elevated RA disease activity with persistent inflammation represents an independent and major risk factor for CVD [50,51].

Outcomes of different experimental studies advocate that physiological levels of TNF-alpha may have beneficial effects on acute heart ischemia. Furthermore, this cytokine plays an important role in heart remodeling and tissue repair capability. TNFi will eventually decrease serum levels of TNF-alpha and prevent its beneficial effects [52-54]. Protection by exogenous TNF-alpha requires a washout phase before sustained ischemia is induced, which suggests that TNF-alpha acts like a trigger of preconditioning [55]. This might be the reason that TNF-alpha antagonism causes an increased risk of heart failure hospitalization. TNF-alpha levels are elevated in patients diagnosed with heart failure due possibly due to an increase in ventricular wall stress. It is also known to cause ventricular remodeling, myocyte fibrosis, and cell death [56,57]. Some studies suggest that TNFi may potentiate cardiotoxicity caused by TNF-alpha. Etanercept has been shown to increase the duration of exposure of cardiac tissue to TNF-alpha, thus increasing the risk of cardiac toxicity [58]. Infliximab has been postulated to cause lysis of a cell in the presence of complements [59]. The combination of side effects that these TNFi portray would ultimately lead to heart failure or exacerbation of pre-existing heart failure. As such, TNFi should be used with caution in patients with heart failure.

Strengths and limitations

Our systematic review used meticulous methods, which included an extensive literature search and risk of bias ascertainment, and two reviewers independently assessed the relevance in an attempt to minimize publishing bias.

Limitations of our study include the expectation that the patients were receiving the prescribed treatment that fit the inclusion criteria of respective treatment groups. Information is not available regarding the proportion of patients that were receiving DMARD therapy or its dosage. We also had to assume that both treatment groups were treated to the same remission goals.

Furthermore, this review only included patients with RA and not other inflammatory arthropathies. This is partly due to the vast body of evidence that RA is associated with an increased risk of CVD. It must be kept in mind that this study may not be generalizable to other inflammatory rheumatic conditions treated with TNFi.

## Conclusions

In conclusion, we identified that RA treatment with TNFi therapy is associated with a reduced risk of cardiovascular events, a finding that is supported by numerous studies that outline its pathophysiology. This shows the potential benefit of TNFi in patients who are diagnosed with RA and have comorbid coronary artery disease and its risk factors such as hyperlipidemia. It is not well known if TNFi monotherapy can affect mortality in patients with RA and this shows a future area of study. We encourage further observation of the effect of TNFi therapy benefits in RA, especially in studies (both cohort and RCTs) that include a longer duration of follow-up and provide more detailed information on potential confounders and the duration of RA.
